# The use of the *ph1b* mutant to induce recombination between the chromosomes of wheat and barley

**DOI:** 10.3389/fpls.2015.00160

**Published:** 2015-03-19

**Authors:** María-Dolores Rey, María C. Calderón, Pilar Prieto

**Affiliations:** Plant Breeding Department, Institute for Sustainable Agriculture, Agencia Estatal Consejo Superior de Investigaciones Científicas Córdoba, Spain

**Keywords:** *Triticum*, *Hordeum* substitution and addition lines, *Ph1* locus, wheat breeding, recombination, meiosis

## Abstract

Intensive breeding has led to a narrowing in the genetic base of our major crops. In wheat, access to the extensive gene pool residing in its many and varied relatives (some cultivated, others wild) is hampered by the block on recombination imposed by the *Ph1 (Pairing homoeologous 1)* gene. Here, the *ph1b* mutant has been exploited to induced allosyndesis between wheat chromosomes and those of both *Hordeum vulgare* (cultivated barley) and *H. chilense* (a wild barley). A number of single chromosome *Hordeum* sp. substitution and addition lines in wheat were crossed and backcrossed to the *ph1b* mutant to produce plants in which pairing between the wheat and the non-wheat chromosomes was not suppressed by the presence of *Ph1*. Genomic *in situ* hybridization was applied to almost 500 BC_1_F_2_ progeny as a screen for allosyndetic recombinants. Chromosome rearrangements were detected affecting *H. chilense* chromosomes 4**H^**ch**^**, 5**H^**ch**^**, 6**H^**ch**^**, and 7**H^**ch**^** and *H. vulgare* chromosomes 4**H^**v**^**, 6**H^**v**^**, and 7**H^**v**^**. Two of these were clearly the product of a recombination event involving chromosome 4**H^**ch**^** and a wheat chromosome.

## Introduction

Bread wheat (*Triticum aestivum*) is one of the most important food crops of the world, and continuous improvement in its productivity will be required to keep pace with global population growth. The genetic base of the species is rather narrow, as its speciation was very recent (Salamini et al., 2002; Riehl et al., 2013). However, a large number of sexually compatible species (some wild and some cultivated) are known, and these represent a much needed reservoir of potentially exploitable genetic variation.

The genome of an interspecific or (intergeneric) hybrid combines the haploid complements of each of its sexual parents. Even though their genomes are closely related to one another, in most cases, the chromosomes of wheat and those of its relatives fail to pair with one another and thus allosyndetic recombination is rare. The failure of homoeologs (chromosomes from related genomes but not completely homologous) to pair at meiosis is ensured by the wild type allele at the *Ph1* locus (Riley and Chapman, 1958; Sears and Okamoto, 1958; Sears, 1976). This gene imposes diploid-like chromosome behavior during meiosis, even though the constituent sub-genomes of this hexaploid species are known to be very closely related to one another. Deletion of the *Ph1* locus allows homoeologs to pair relatively freely with one another (Moore, 2014), a situation which has been exploited for introgression purposes through the use of the *ph1b* mutant (Riley et al., 1968b; Sears, 1977, 1981, 1982; Khan, 1999; Lukaszewski, 2000; Qi et al., 2008; Liu et al., 2011; Zhao et al., 2013).

*Hordeum chilense*, a species which is readily crossable with wheat, is a diploid relative of cultivated barley. It has been identified as a potential donor to wheat for a number of traits of agronomic interest (Martín et al., 1998, 2000). The bread wheat × *H. chilense* hybrid has been the source of a collection of single (*Hordeum*) chromosome addition lines and chromosome substitution lines in a bread wheat genetic background (Miller et al., 1982), and similar cytogenetic stocks have been developed involving the cultivated barley (*H. vulgare*) chromosomes (Islam et al., 1978, 1981). The self-fertile amphidiploid *Tritordeum* represents the product of chromosome doubling of the hybrid *T. turgidum* × *H. chilense* (Martín and Sanchez-Mongelaguna, 1982). The presence of *Ph1* maintains the integrity of *Hordeum* sp. chromosome(s) in all of this germplasm, meaning that the introgression of favorable non-wheat genes is inevitably accompanied by the inheritance of a large number of unwanted ones. The experience with introgression into wheat from other related species suggests that this linkage drag can best be overcome by employing a *ph1b*-based strategy. Here, we describe progress made with an introgression program using the *ph1b* mutant to induce chromosome pairing and recombination between the chromosomes of *H. chilense* or *H. vulgare*, and those of wheat.

## Materials and Methods

### Plant Materials

**Table 1** lists the various *H. chilense* substitution lines and *H. chilense* and *H. vulgare* addition lines (Islam et al., 1978, 1981; Miller et al., 1982) used as the female parent in crosses with the *ph1b* mutant (Sears, 1977). Grains were germinated on wet filter paper in the dark for 5 days at 4^∘^C, followed by a period of 24 h at 25^∘^C. Emerging seedling roots were excised, incubated for 4 h in 0.05% w/v colchicine at 25^∘^C, fixed in Carnoy’s solution (three parts 100% ethanol plus one part glacial acetic acid), and finally stored at 4 C for at least 1 month. The plants were subsequently raised in a greenhouse held at 26 C during the day and 22^∘^C during the night (16 h photoperiod). Immature spikes were fixed in Carnoy’s solution and used to characterize chromosome pairing at meiosis metaphase I.

**Table 1:** Plants used for crosses made to engineer individuals carrying a *Hordeum* sp. chromosome in a *ph1b* mutant background.

Initial parental lines				Descendence		
Wheat line (female), nomenclature and number of plants used			CS*ph1ph1* (male)	F1	BC1F1	BC1F2
(4**B**)4**H^**ch**^** disomic substitution line	CS(4**B**)4**H^**ch**^**	5	3	15	17	30
(4**D**)4**H^**ch**^** disomic substitution line	CS(4**D**)4**H^**ch**^**	5	3	11	15	48
(5**A**)5**H^**ch**^** disomic substitution line	CS(5**A**)5**H^**ch**^**	5	3	16	22	12
(5**B**)5**H^**ch**^** disomic substitution line	CS(5**B**)5**H^**ch**^**	5	3	15	48	30
(5**D**)5**H^**ch**^** disomic substitution line	CS(5**D**)5**H^**ch**^**	5	3	11	22	20
(7**A**)7**H^**ch**^** disomic substitution line	CS(7**A**)7**H^**ch**^**	5	3	21	47	77
(7**B**)7**H^**ch**^** disomic substitution line	CS(7**B**)7**H^**ch**^**	5	3	19	59	64
(7**D**)7**H^**ch**^** disomic substitution line	CS(7**D**)7**H^**ch**^**	5	3	19	36	40
5**H^**ch**^** disomic addition line	5**H^**ch**^** addition	5	3	5	27	–
6**H^**ch**^** disomic addition line	6**H^**ch**^** addition	5	3	29	35	20
7**H^**ch**^** disomic addition line	7**H^**ch**^** addition	5	3	16	21	25
**Total of wheat-*H. chilense* plants**		**55**	**33**	**177**	**349**	**366**
2**H^**v**^** disomic addition line	2**H^**v**^** addition	5	3	11	20	–
4**H^**v**^** disomic addition line	4**H^**v**^** addition	5	3	15	52	46
6**H^**v**^** disomic addition line	6**H^**v**^** addition	5	3	23	33	23
**7H^**v**^** disomic addition line	7**H^**v**^** addition	5	3	21	28	38
**Total of wheat-*H.vulgare* plants**		**20**	**12**	**70**	**133**	**107**
**Total**		**75**	**45**	**218**	**482**	**473**

*CS, wheat cv. Chinese Spring; **H^**ch**^**, *H. chilense*; **H^**v**^**, H. vulgare.*

### DNA Marker Characterization

Genomic DNA was extracted from frozen seedling leaves following Murray and Thompson (1980), as modified by Hernández et al. (2001). The absence of *Ph1* was verified using a PCR assay described by Wang et al. (2002). Each 30 μL PCR contained 1x PCR buffer with MgCl_2_ (Bioline USA, Taunton, MA, USA), 0.25 mM dNTP, 0.17 μM primers, 0.02 U/μL Taq DNA polymerase (Bioline USA), and 20 ng template. The reaction was first denatured (94^∘^C/5 min), and then subjected to 35 cycles of 94^∘^C/60 s, 51^∘^C/60 s, and 72^∘^C/60 s, followed by a final extension (72^∘^C/7 min). The PCR products were electrophoretically separated through a 1% agarose gel and visualized by EtBr staining. The presence of each *Hordeum* sp. chromosome was based on PCR assays described by Liu et al. (1996) and Hagras et al. (2005) as detailed in **Table 2**. The composition of these PCR reactions was as above, while the amplification regime comprised an initial denaturing step (94^∘^C/5 min), followed by 35 cycles of 94^∘^C/15 s, 50–65^∘^C (primer dependent, see **Table 2**) /30 s, 72^∘^C/60 s, and completed by a final extension (72^∘^C/6 min). The amplicons were separated as described above.

**Table 2:** DNA-based markers used as genotypic assays for the presence of specific *Hordeum* sp. chromosomes.

Marker name	Sequence of primers (5‘→3^′^)	*Hordeum* chromosome	Annealing temperature (^∘^C)
BAWU759-F	TCGACATCTCTCCCATTTCCC	2**H**-S	50
BAWU759-R	AACCAGATATGGATGCCAGG	2**H**-S	50
HVCSG-F*	CACTTGCCTACCTCGA TAAGTTTGC	2**H^**v**^ -** L	50
HVCSG-R*	GTGGATTCCATGCATGCA ATATGTGG	2**H^**v**^ -** L	50
BAWU303-F	AATGTGCCTCCACAGGGTAG	4**H**-S	55
BAWU303-R	GATACTGAGTGGAAAGCGGC	4**H**-S	55
BAWU808-F	TGCCCCCAAACTTTATATGC	4**H**-L	55
BAWU808-R	GAGGGTCTTCCTGTTGTGGA	4**H**-L	55
BAWU131-F	GAACGCCAGCCAAATTGTAT	5**H**-S	60
BAWU131-R	ACCATTTTGATCCTTCTGCG	5**H**-S	60
BAWU782-F	CAACTTGGACAACACAACGC	5**H**-L	60
BAWU782-R	CTTGTGCATGCGCAGAGTAT	5**H**-L	60
BAWU94-F	TTTCAAGCAGAGCTGCAAAG	6**H**-S	55
BAWU94-R	GCTTGCTGAGCGCTTTCTAC	6**H**-S	55
BAWU107-F	CGCCTATTTCTGAGCTCCTG	6**H**-L	55
BAWU107-R	CGAGTATGGGAGTGGCAGTT	6**H**-L	55
BAWU763-F	AGAACCGAGATGAGGAATGTG	7**H**-S	58
BAWU763-R	AGTCTCTTCGCGGAATCAAG	7**H**-S	58
BAWU550-F	ATGCCACCATTTACAAAGCC	7**H**-L	50
BAWU550-R	TTTCTGGGTCCTGATCCTTG	7**H**-L	50

F, Forward primer; R, reverse primer; **H^**v**^**, *H. vulgare*; **H**, *H. chilense* and *H. vulgare*.

### Cytogenetic Analysis

Chromosome spreads were prepared from both pollen mother cells (PMCs) at meiotic metaphase I and from root tip cells. The material was macerated in a drop of 45% glacial acetic acid, squashed under a cover slip, and dipped in liquid nitrogen in order to remove the cover slip. The preparations were then air-dried and either processed directly for *in situ* hybridization, or stored at 4^∘^C until required. The probe used for genomic *in situ* hybridization was genomic DNA extracted from *H. chilense* (or *H. vulgare*) seedling leaves. The DNA was labeled with either biotin-11-dUTP (*H. vulgare*) or digoxigenin-11-dUTP (*H. chilense*; both from Roche Corporate, Basel, Switzerland) by nick-translation. The *in situ* hybridization protocol followed that described by Prieto et al. (2004). The GAA-satellite sequence (Pedersen et al., 1996) and the pAs1 probe (Rayburn and Gill, 1986) were used to identify chromosomes involved in homoeologous pairing, chromosomal translocations, or chromosomal rearrangements. The GAA-satellite sequence identifies all the A and B wheat chromosomes (Pedersen and Langridge, 1997), whereas the pAs1 identifies the D wheat and the *H. chilense* chromosomes (Cabrera et al., 1995). The GAA-satellite sequence and the pAs1 probes were also labeled by nick translation with biotin-11-dUTP and digoxigenin-11-dUTP, respectively. Biotin- or digoxigenin-labeled DNA were detected using, respectively, streptavidin-Cy3 (Sigma, St. Louis, MO, USA) and antidigoxigenin-FITC (Roche Applied Science, Indianapolis, IN, USA). After counter-staining with DAPI (4^′^, 6-diamidino-2-phenylindole), the preparations were mounted in Vectashield (Vector Laboratories, Burlingame, CA, USA). Hybridization signals were visualized using a Nikon Eclipse 80i epifluorescence microscopy, and the images captured with a CCD camera (Nikon Instruments Europe BV, Amstelveen, The Netherlands).

### Statistical Methods

Statistical analyses were performed using the STATISTIX v9.0 software (Analytical Software, Tallahassee, FL, USA). Wilcoxon (or *U* of Mann–Whitney) test was used to determine the statistical significance of differences between means.

## Results

### Converting the Substitution and Addition Lines into a *ph1b* Mutant Background

**FIGURE 1:**
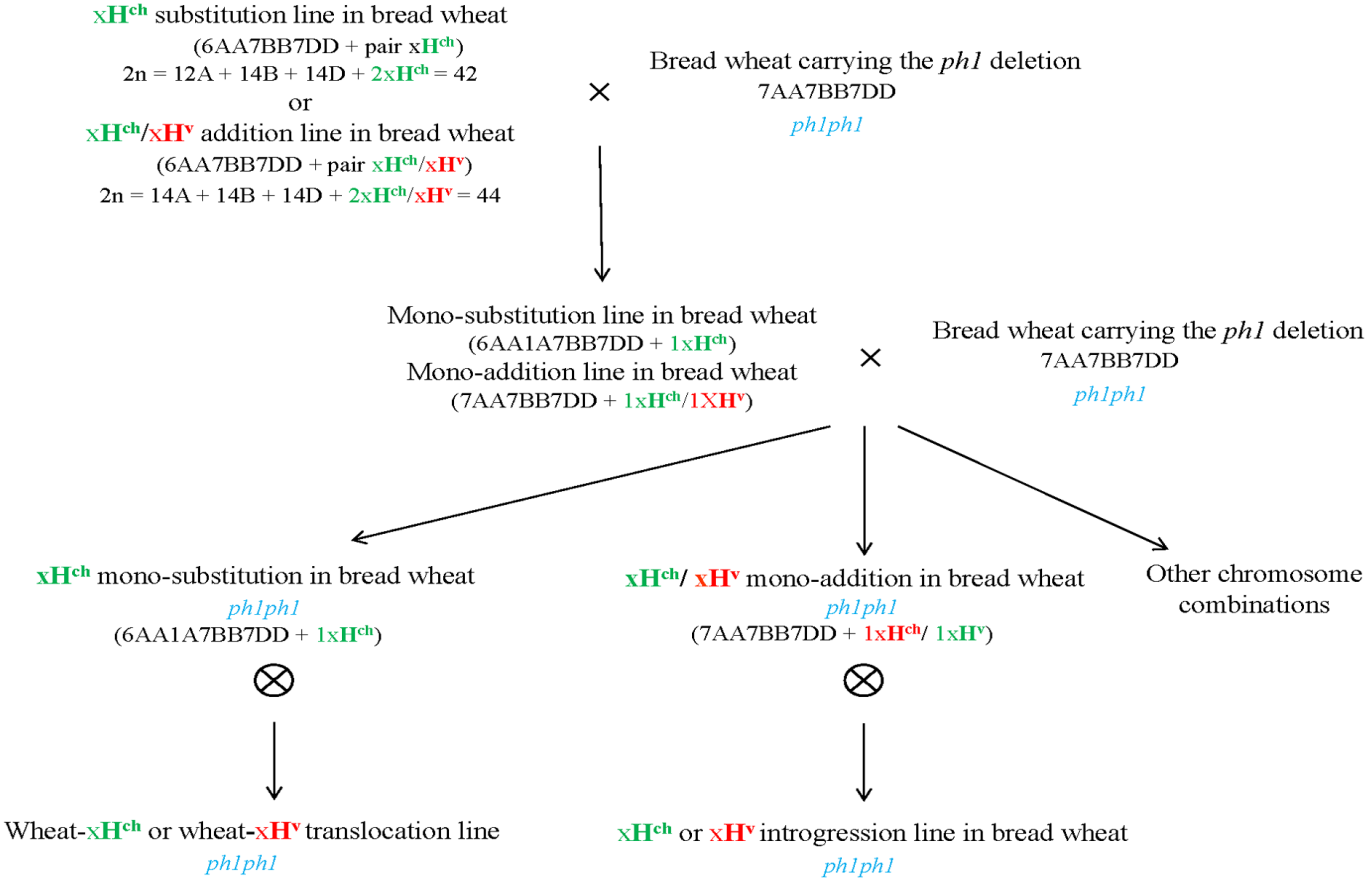
Development of *Hordeum* sp. introgression lines in hexaploid wheat in the *ph1b* mutant background. Crosses between a *Hordeum* sp. substitution or addition line in bread wheat cv. Chinese Spring (2*n* = 6*x* = 42) and the *ph1b* mutant in hexaploid wheat were developed and backcrossed to the *ph1b* mutant to obtain *Hordeum* sp. introgressions in the absence of the *Ph1* locus. Screening and characterization of chromosome complements were carried out by multicolor *in situ* hybridization and molecular markers analyses.

**FIGURE 2:**
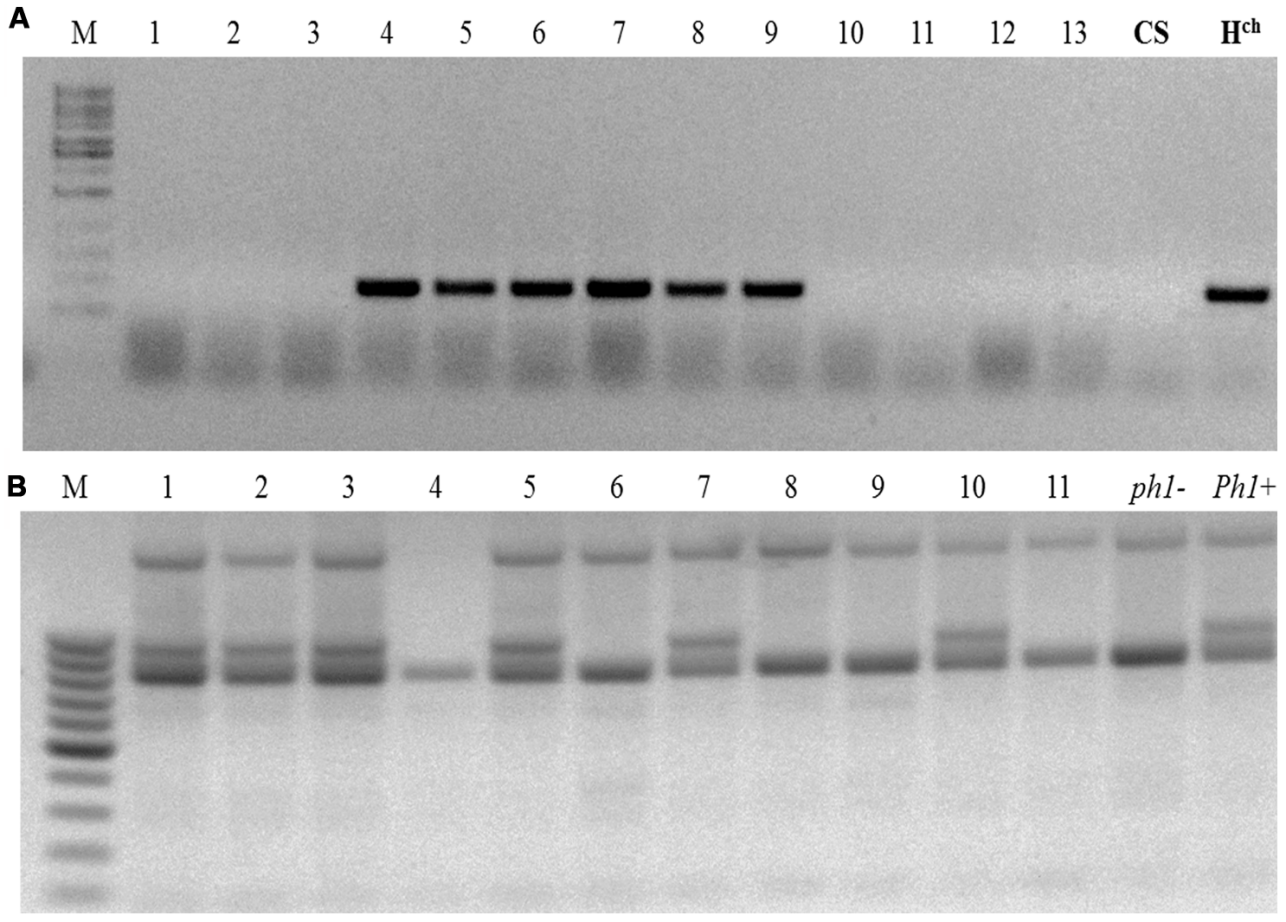
Genotypic assays for the presence of *Ph1* and a *Hordeum* sp. chromosome. **(A)** The presence of chromosomes 4**H^**ch**^** (lanes #4–#9) is marked by the successful amplification of the BAWU303 EST fragment. **(B)** The absence of *Ph1* is marked by the loss of the ABC_920_ SCAR marker (individuals #4, #6, #8, #9, and #11) M: size marker; *ph1-*: the parental *ph1b* mutant, *Ph1+*: wild type wheat. CS, *Triticum aestivum* cv. Chinese Spring; **H^**ch**^**, *Hordeum chilense*.

The crossing scheme used is illustrated in **Figure 1**, and the details of the crossing outcomes from the F1 to the BC_1_F_2_ generation are given in **Table 1**. The F_1_ hybrid progeny were genotyped by PCR to ensure that they had retained the expected *Hordeum* sp. chromosome (**Table 2**; **Figure 2A**), then crossed again to the *ph1b* mutant in order to establish individuals in which the *Hordeum* sp. chromosome was now present in a *ph1bph1b* background. Zygosity at the *Ph1* locus was predicted using a PCR assay (**Figure 2B**). The meiotic behavior of the selected individuals was characterized by GISH analysis of metaphase I in PMCs, and the plants were allowed to self-pollinate.

### Allosyndetic Pairing in BC_1_F_1_ Selections Lacking *Ph1*

**FIGURE 3:**
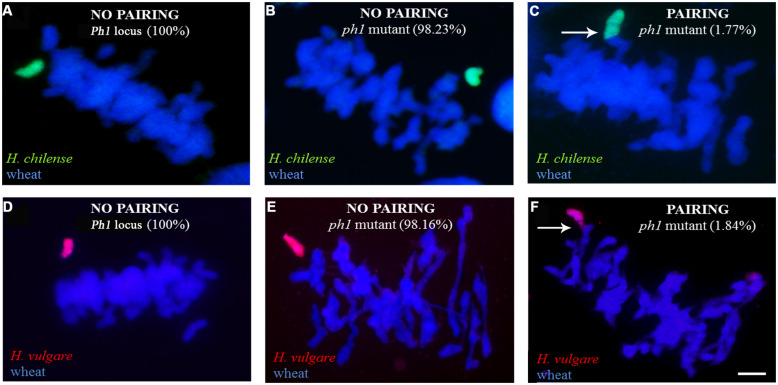
Chromosome pairing at meiotic metaphase I as determined by the allelic status at *Ph1*. In the presence of *Ph1*, the *Hordeum* sp. chromosomes [**(A)** 7**H^**ch**^**, shown in green and **(D)** 4**H^**v**^**, shown in red] remained unpaired. In a *ph1b* background, the *Hordeum* sp. chromosome [**(B)** 5**H^**ch**^**, shown in green and **(E)** 7**H^**v**^**, shown in red] remained as a univalent in most cells. Allosyndesis is induced by the absence of *Ph1* between a *Hordeum* sp. chromosome [**(C)** 5**H^**ch**^**, shown in green and **(F)** 7**H^**v**^**, shown in red], and a wheat chromosome. Arrows indicate pairing between *Hordeum* sp.-wheat homoeologs induced by the absence of the *Ph1* locus. Bar: 10 μm.

**FIGURE 4:**
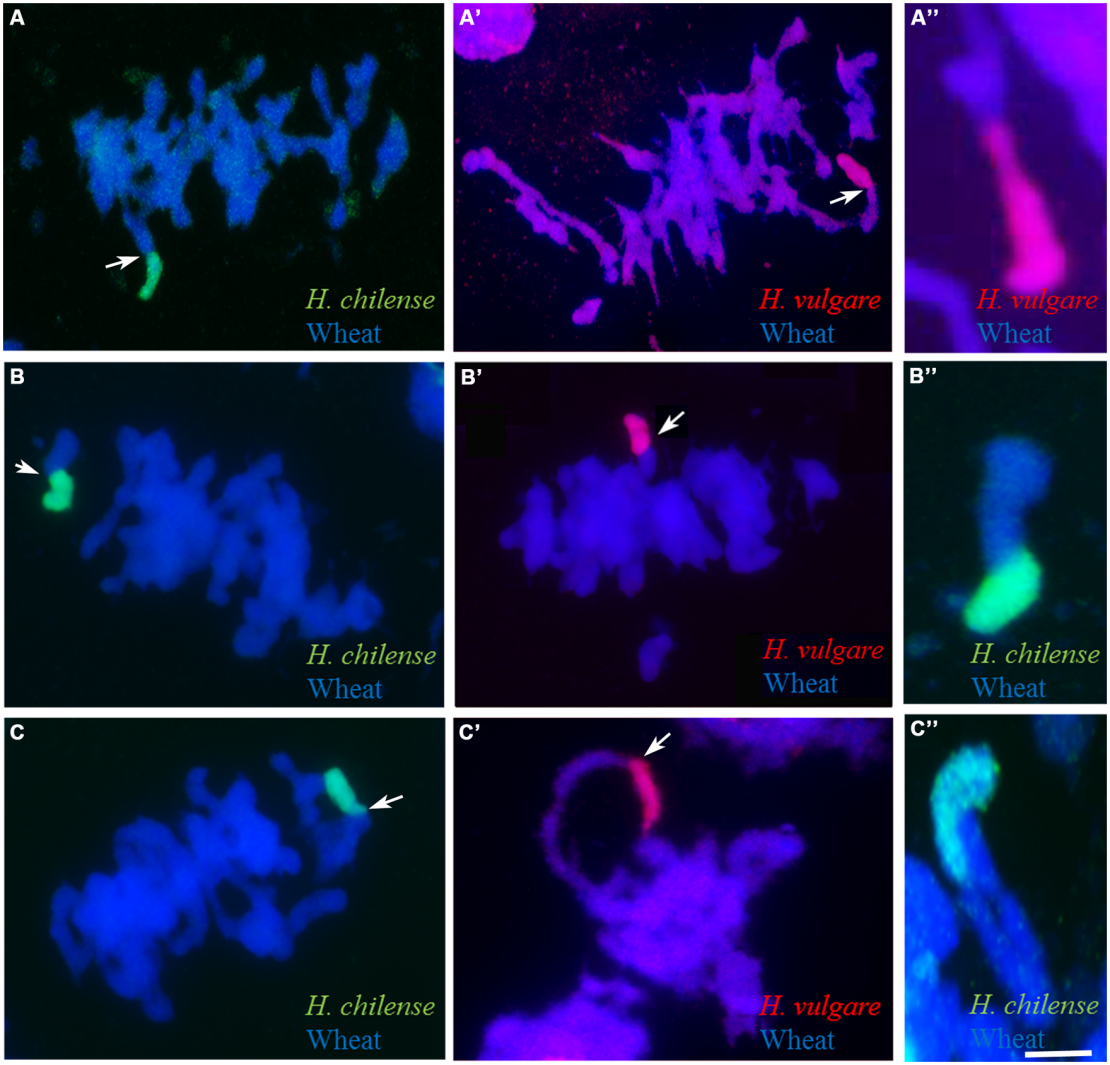
**Hordeum sp./wheat chromosome pairing at meiotic metaphase I as detected by GISH. (A-A")** Rod bivalents with a sub-terminal chiasma. (**B-B"**) Rod bivalents with a more proximal chiasma. (**C-C"**) A Hordeum sp. chromosome involved in a multivalent. Bar: 10 μm.

Meiosis was characterized in 63 BC_1_F_1_ segregants carrying a *Hordeum* chromosome in the absence of *Ph1* and compared to those carrying the *Hordeum* chromosome in its presence (**Table 3**). No wheat/*Hordeum* chromosome pairing occurred in plants of genotype *Ph1Ph1* (**Table 3**; **Figures 3A,D**). In contrast, in the absence of the *Ph1* locus, although the *Hordeum* chromosomes remained unpaired in most metaphase I PMCs (**Figures 3B,E**), pairing was observed in 1.77% of the PMCs in *H. chilense* (**Table 3**; **Figure 3C**). The equivalent frequency with respect to *H. vulgare* chromosomes was 1.84% (**Table 3**; **Figure 3F**). The frequency of plants displaying wheat/*Hordeum* chromosome associations was lower in *H. chilense* than in *H. vulgare* (45.23% and 61.90%, respectively), although variability depending on the specific *Hordeum* sp. chromosome introgressed was found. Most of the associations between a *Hordeum* and a wheat chromosome involved the formation of a rod bivalent harboring a single sub-terminal chiasma (**Figures 4A-A”**), although in some cases the chiasma occurred more proximally (**Figures 3B,B”**). In a few PMCs, the *Hordeum* sp. chromosome formed part of a multivalent (**Figures 4C-C”**) as the result of chiasmata between homoeologous chromosomes, or reflecting the re-arrangement of the wheat genome induced by successive meiosis during the generations of selfing used to maintain the *ph1b* mutant stock. Wilcoxon test showed that the frequency of allosyndesis was not *Hordeum* sp. chromosome specific, since there was no significant difference in pairing frequency between either chromosomes 4**H^**ch**^**, 6**H^**ch**^**, and 7**H^**ch**^** or between chromosomes 4**H^**v**^**, 6**H^**v**^**, and 7**H^**v**^** (**Table 4A**). In addition, using the same statistical test, no significance differences where found when compared the effect of the genome (*H. chilense* or *H. vulgare*) for the same homoeologous group (*p* = 0.39, 0.41, and 0.70 for chromosomes 4, 6, and 7, respectively; **Table 4A**). A statistical comparison of chromosome pairing frequency involving a *H. chilense* chromosome and each of its wheat homoeologs was also carried out and showed no evidence for any preferential pairing (**Table 4B**).

**Table 3:** The frequency of allosyndesis involving a *Hordeum* and a wheat chromosome in either the presence (*Ph1+*) or absence (*ph1-*) of the *Ph1* locus.

Wheat line	*Hordeum* sp. introgressed	No of plants analyzed	No of plants showing wheat-*Hordeum* pairing	Frequency of wheat-*Hordeum* pairing (%)	No of PMCs scored	No of PMCs scored showing wheat-*Hordeum* pairing	Frequency of wheat-*Hordeum* pairing in PMCs (%)	*p*-value
*Ph1^+^*		5	0	0.00	206	0	0.00	*p =* 0.000^∗∗∗^
*ph1-*	*H. chilense*	42	19	45.23	2422	43	1.77	
	*H. vulgare*	21	13	61.90	1352	25	1.84	
	Total	63	32	53.56	3774	67	1.80	


**Table 4:** **(A)** The frequency of allosyndesis between individual *H. chilense* or *H. vulgare* chromosomes and those of wheat. **(B)** The frequency of pairing between specific *Hordeum* chromosomes and each of their wheat homoeologs.

(A) Frequency of *Hordeum*-wheat pairing (%)
**Genome**	**Chromosome 4**	**Chromosome 6**	**Chromosome 7**	***p*-value**

*H. chilense*	1.59	1.65	1.83	0.63 (p>0.05)
*H. vulgare*	1.24	2.78	0.86	0.75 (p > 0.05)

*p*-value	0.39 (p > 0.05)	0.41 (p > 0.05)	0.70 (p > 0.05)	

**(B) Frequency of** ***Hordeum*-wheat pairing (%)**

**Wheat homoeology group**	**Chromosome 4H**	**Chromosome 5H**	**Chromosome 7H**

A	–	3.55	0.79
B	0.31	2.85	2.78
D	2.87	2.68	4.09

*p*-value	0.37 (p > 0.05)	0.42 (p > 0.05)	0.30 (p > 0.05)

### Genetic Evidence for *Hordeum* sp. Introgression Induced by the Absence of *Ph1*

**FIGURE 5:**
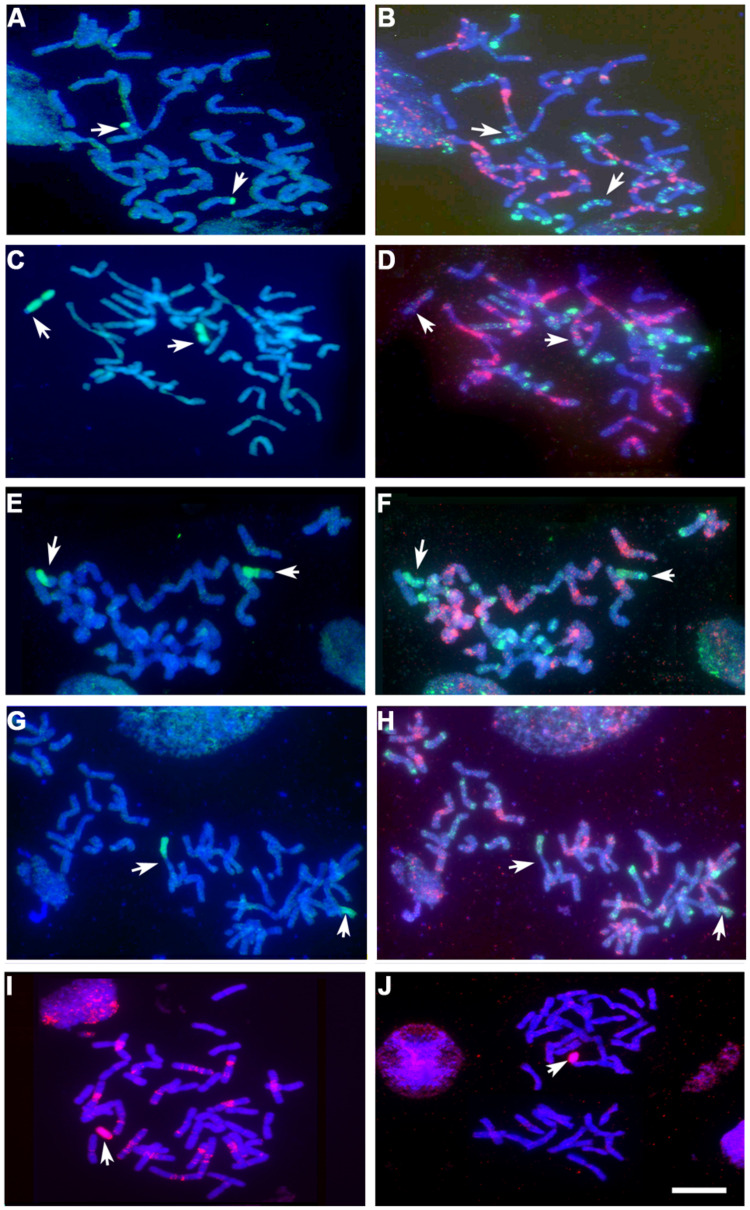
Various forms of introgression (arrowed) detected by GISH (*Hordeum chilense* and *H. vulgare* genomic introgressions detected in green and red, respectively), and FISH patterns (GAA and pAs1 probes detected in red and green, respectively), in the BC_1_F_2_ progeny derived from the crosses *Hordeum* addition/substitution line × *ph1b* mutant. **(A)** GISH and **(B)** FISH pattern of chromosomes of a partial mitotic metaphase carrying two copies of a 4**D** chromosome with a distal 4**H^**ch**^** L segment. **(C)** GISH and **(D)** FISH pattern of a mitotic metaphase carrying two copies of a 4**H^**ch**^** chromosome with a distal 4**D** L segment. **(E)** GISH and **(F)** FISH pattern of a mitotic metaphase carrying a homocigous 7**H^**ch**^** S–7**A** L Robertsonian translocation.** (G)** GISH and **(H)** FISH pattern of a mitotic metaphase carrying a homocigous 7**A** S–7**H^**ch**^** L Robertsonian translocation. **(I)** GISH of a 4**H^**v**^** monotelosomic line. **(J)** GISH of a 6**H^**v**^** monotelosomic line. Bar: 10 μm.

A total of 473 BC_1_F_2_ progeny were analyzed by GISH analysis to detect and characterize *Hordeum* sp. chromosome re-arrangements in the background of the *ph1b* mutant. About 60% of the progeny lacked any *Hordeum* sp. chromatin. Overall, with respect to the *Hordeum* sp. chromosome, about 3% of the progeny were disomic and about 33% were monosomic. The highest transmission rate of a *Hordeum* chromosome was observed among the progeny derived from the (4**B**) 4**H^**ch**^** substitution line. Two recombinants were identified, both involving chromosomes 4**H^**ch**^** and 4**D** (**Table 5**; **Figures 5A-D**). A total of 15 individuals harbored a Robertsonian translocation involving a *H. chilense* (chromosome 5**H^**ch**^**: one plant, chromosome 7**H^**ch**^**: 14 plants) and the homoeologous wheat chromosomes 5**B** and 7**A**, respectively (**Table 5**; **Figures 5E-H**). Telosomic chromosomes resulting from misdivision were observed in eight plants, affecting chromosomes 6**H^**ch**^**, 7**H^**ch**^**, 4**H^**v**^**, 6**H^**v**^**, and 7**H^**v**^** (**Table 5**; **Figures 5I,J**).

**Table 5:** BC_1_F_2_ progeny retaining *H. chilense* or *H. vulgare* chromatin.

Wheat line	No of plants					
	Complete chromosome			*Hordeum*-wheat translocations	Telosomic chromosome	Small introgression	Total


	2 copies	1 copy	0 copies				
**CS(4B)4H^**ch**^**	2	13	15	0	0	0	30
**CS(4D)4H^**ch**^**	0	15	31	0	0	**2 (4.2%)**	48
**CS(5A)5H^**ch**^**	0	5	7	0	0	0	12
**CS(5B)5H^**ch**^**	1	10	18	**1 (3.3%)**	0	0	30
**CS(5D)5H^**ch**^**	0	5	15	0	0	0	20
**CS(7A)7H^**ch**^**	2	32	37	**5 (6.3%)**	1	0	77
**CS(7B)7H^**ch**^**	1	20	37	**4 (6.2%)**	2	0	64
**CS(7D)7H^**ch**^**	0	11	26	**3 (7.5%)**	0	0	40
**6H^**ch**^ addition**	0	8	11	0	1	0	20
**7H^**ch**^ addition**	2	6	15	**2 (8%)**	0	0	25
**4H^**v**^ addition**	3	14	28	0	1	0	46
**6H^**v**^ addition**	2	9	11	0	1	0	23
**7H^**v**^ addition**	1	10	25	0	2	0	38
**Total**	14	158	276	15	8	2	**473**


Wheat plants carrying stable chromosome introgressions are in bold.

## Discussion

Interspecific hybridization retains its potential to widen the gene pool available to the wheat breeder. Combining *in situ* hybridization with DNA-based genotyping has eased the process considerably since the initial efforts which followed the recognition that recombination could be induced by the deletion of *Ph1* (Koebner and Shepherd, 1986; Qi et al., 2007). An *in situ* hybridization-based screening strategy has previously been applied to characterize introgressions from both *H. chilense* and *H. vulgare*, resulting in the recognition of a number of wheat/*Hordeum* sp. translocations (Prieto et al., 2001). Here, the intention was to exploit the abolition of strict homologous pairing induced by the absence of *Ph1* to generate material where recombination had shortened the length of the introgressed segment. Chromosome 4**H^**ch**^** is of particular interest as it harbors a gene (or possibly genes) encoding resistance against the fungal pathogen *Septoria tritici* (Rubiales et al., 2000). Two recombinants involving chromosome 4**H^**ch**^** were obtained in this work as the results of the same recombination event between 4**D**L and 4**H^**ch**^**L chromosome arms, and can help to locate those resistance genes on chromosome 4**H^**ch**^** L. Similarly, chromosome 7**H^**ch**^** has been targeted for its positive effect on grain carotenoid content (Alvarez et al., 1999), and chromosome 5**H^**ch**^** for its contribution to enhancing salinity tolerance (Forster et al., 1990). Although inter-chromosome translocations are known to occur spontaneously (Mettin et al., 1973; Zeller, 1973; Prieto et al., 2001), and can be induced by ionizing radiation and the action of certain gametocidal genes (Sears, 1956, 1993; Endo, 1988, 1990; Endo and Gill, 1996), the particular advantage of exploiting the *ph1b* mutant to promote allosyndesis is that the translocations are non-random: rather, they tend to involve the exchange of genetically related material. Its disadvantage is that the frequency of allosyndesis (and hence of recombination) is rather low, especially between chromosomes of more distantly related genomes such as *Triticum* and *Hordeum.* The level of *ph1b-* induced pairing between wheat and cereal rye (*Secale cereale*) chromosomes has been estimated to be around 4% (Miller et al., 1994), which is about double the level noted here between the chromosomes of wheat and either of the two *Hordeum* sp. Moreover, the frequency of recombination was correlated with the frequency of wheat-rye pairing in metaphase I in ABDR hybrids in the absence of the *Ph1* locus (Naranjo and Fernández-Rueda, 1996). However, an extensive *ph1b*-based attempt to reduce the length of the rye chromosome segment present in the widely used wheat/rye Robertsonian translocation 1**B**L.1**R**S resulted in an estimated recombination frequency of only around 0.7% (Koebner and Shepherd, 1986; Lukaszewski, 2000). The levels achievable in more closely related species, notably in the genus *Aegilops* (Riley et al., 1968a; Gill and Raupp, 1987; Koebner and Shepherd, 1987; Farooq et al., 1990; Ceoloni et al., 1992), are much higher than this.

Our results showed that homoeologous recombination between *Hordeum* sp. and wheat chromosomes did only depend on the absence of the *Ph1* locus as no differences in the frequency of pairing were found when chromosome association in different homoeologous groups was studied. Most of chromosome associations between *Hordeum* sp. and wheat chromosomes were end-to-end extremely distal associations as described previously (Werner et al., 1992; Benavente et al., 1996; Calderón et al., 2014).

In summary, the use of the *ph1b* mutant does induce a low, but significant level of chromosome pairing and recombination between wheat and *Hordeum* sp. chromosomes. The translocation and introgression chromosomes detected in the present work will serve as potential donor material for the breeding of cultivars having a higher grain carotenoid content, stronger resistance against *S. tritici* and improved salinity tolerance.

## Author Contributions

M-DR, MC, and PP carried out the experiments and analyzed the data. M-DR and PP planned the study and wrote the manuscript. All authors read and approved the final manuscript.

## Conflict of Interest Statement

The authors declare that the research was conducted in the absence of any commercial or financial relationships that could be construed as a potential conflict of interest.
